# Prevalence of Obsessive-Compulsive Symptoms Among University Students in Tabuk: A Cross-Sectional Study

**DOI:** 10.7759/cureus.87466

**Published:** 2025-07-07

**Authors:** Sami Assil, Naif A Alghamdi, Saud A Alghamdi

**Affiliations:** 1 Preventive Medicine, King Fahad Specialist Hospital, Tabuk, SAU; 2 Psychiatry, Ministry of Health Holdings, Tabuk, SAU; 3 Dentistry, University of Al Baha, Al Baha, SAU

**Keywords:** cross sectional studies, ocd, prevalence, psychiatry, psychology, saudi arabia, university of tabuk

## Abstract

Background: Obsessive-compulsive disorder (OCD) is a prevalent psychiatric disorder that can significantly impair functioning and well-being. While OCD has been extensively studied in various populations, data on its prevalence among university students in the Tabuk region of Saudi Arabia remain limited.

Objective: To determine the prevalence of obsessive-compulsive symptoms among students at Tabuk University and explore their association with demographic and academic characteristics.

Methods: A cross-sectional study was conducted from August 2024 to December 2024 among 190 students at Tabuk University. Data were collected using an electronic questionnaire including socio-demographic information and the Arabic version of the Obsessive-Compulsive Inventory-Revised (OCI-R). Statistical analysis was performed using IBM SPSS Statistics, Version 30.0.

Results: Among the participants, 41.6% showed probable OCD symptoms. Female students demonstrated a higher prevalence (45.3%) compared to males (36.9%). Students living alone or with roommates showed higher rates of probable OCD symptoms (54.2% and 50.0%, respectively) compared to those living with family (39.4%). Engineering and applied medical sciences students showed the highest rates of probable OCD symptoms (58.3% and 57.1%, respectively), while medical students showed the lowest (25.8%). Fifth-year students demonstrated the highest prevalence (77.8%).

Conclusion: A considerable proportion of Tabuk University students exhibit probable OCD symptoms, with variations across different demographic and academic characteristics. These findings highlight the need for targeted mental health interventions and support services within the university setting.

## Introduction

Obsessive-compulsive disorder (OCD) is a mental health condition characterized by recurring intrusive thoughts, images, or impulses (obsessions) and repetitive behaviors or mental acts (compulsions). These symptoms can significantly disrupt daily functioning and cause considerable distress [1]. In the United States, OCD affects approximately 2-3% of the population, with a slightly higher prevalence among women than men. While OCD typically emerges during childhood, adolescence, or early adulthood, it can develop at any age [2].

Research has identified excessive worry as a significant factor in the development of OCD symptoms, highlighting its role in the disorder's pathogenesis [3]. Additionally, OCD frequently co-occurs with other conditions, particularly anxiety disorders and depression [4]. A research study involving 2,073 participants found that over 25% had experienced obsessions or compulsions at some point in their lives. However, only 2.3% met the full diagnostic criteria for OCD at any time in their lives, and just 1.2% met the criteria within the past year. The study also found that individuals who experienced more frequent or intense obsessions and compulsions were more likely to be diagnosed with OCD. Furthermore, OCD was strongly linked to other mental health conditions, including anxiety and mood disorders, impulse control issues, and substance use disorders [5].

Individuals with OCD often experience limitations in their psychosocial and occupational functioning, which negatively impacts their overall quality of life. These impairments affect various aspects of daily life, including interpersonal relationships, routine activities, and work performance [6]. A cross-sectional study conducted among medical students in Riyadh, Saudi Arabia, investigated the prevalence of OCD. The study found that 36.2% of the student population had OCD, with significantly higher rates among female students and those with depression [7].

A study conducted in the southern region of Saudi Arabia investigated the characteristics of individuals with OCD (n=1,000). Among these individuals, 58.8% were females, while 41.2% were males. Approximately 76.5% of the subjects were single. In terms of income, the majority of the group (61.8%) reported having sufficient monthly income. Furthermore, 70.6% of the subjects had a university education level, indicating a higher prevalence of OCD symptoms among individuals with higher levels of education [8].

At Taibah University in Saudi Arabia, a study exploring the prevalence of OCD among medical students found that 26% of the 263 participants were likely to have OCD [9]. Similarly, during the COVID-19 pandemic [10], a study involving 1,644 undergraduate medical students in Iraq revealed that 67.9% of participants were female, and 70.1% reported experiencing additional mental health symptoms. The study also found that 43% of the students exhibited symptoms indicative of probable OCD, suggesting a pressing need for further mental health assessment and intervention.

Acknowledging the elevated prevalence of OCD observed within our community and suspecting a similar prevalence among students at Tabuk University, the aim of this study is to determine the prevalence of OCD among students enrolled at Tabuk University.

## Materials and methods

Study design, area, and period

The study was conducted at Tabuk University in Tabuk City, Saudi Arabia. A cross-sectional study was carried out from August 2024 to December 2024 to assess the prevalence of OCD among Tabuk University students.

Study population

The study included students enrolled at Tabuk University who met specific inclusion and exclusion criteria. Eligible participants were currently enrolled students from preparatory year through seventh year, including both male and female students who provided voluntary informed consent. Students excluded from the study were those not enrolled at Tabuk University, those under 18 years of age, and those who declined to participate.

Sample size and sampling

Using G Power software with an effect size of 0.5, an alpha error of 0.05, and a power of 0.95, the required sample size was calculated as 176 participants. The study employed convenience sampling and successfully recruited 190 participants [11].

Data collection tools

Participants were recruited through a combination of in-person outreach at the university and online distribution via social media platforms commonly used by students (e.g., WhatsApp, X app, and Telegram). This approach helped ensure a broader reach and participation across different academic levels and disciplines.

Data were collected using a structured electronic questionnaire via Google Forms. The survey instrument was validated through expert consultation and showed high reliability (Cronbach's α=0.89). The questionnaire gathered socio-demographic information (including age, gender, residence, academic year, nationality, marital status, and monthly income) and utilized the Arabic version of the Obsessive-Compulsive Inventory-Revised (OCI-R) from a previous study with permission [9].

The OCI-R consisted of 18 items with answers on a five-point Likert scale (not at all, a little, moderately, a lot, and extremely), coded from 0 to 4, respectively (see Table 5 in the Appendix). Total scores ranged from 0 to 72, with scores above 21 indicating probable OCD symptoms. This validated Arabic version was used to evaluate participants' OCD symptoms [12-14]. The internal consistency of the OCI-R was assessed using Cronbach's alpha coefficient.

Ethical considerations

The study received approval from the Research Ethics Committee of Tabuk Health Affairs. All participants provided informed consent. Data confidentiality was maintained through comprehensive measures, including password-protected electronic storage, restricted access to authorized research personnel, data anonymization and coding, separation of identifying information from research data, and strict compliance with data protection regulations.

Statistical analysis

Data analysis was performed using IBM SPSS Statistics for Windows, Version 30.0 (Released 2024; IBM Corp., Armonk, New York, United States). Descriptive statistics were calculated for all variables, with frequencies and percentages reported for categorical variables. Chi-square tests were employed to examine associations between categorical variables. Fisher's exact test was utilized for cells with expected frequencies less than 5. Statistical significance was set at p<0.05 (two-tailed).

## Results

A total of 190 students participated in this cross-sectional study. Table 1 presents the demographic characteristics of the participants. The majority of students were under 21 years of age, with 111 participants (58.4%). Female students comprised 106 (55.8%) of the sample, while male students accounted for 84 (44.2%). Most participants were unmarried, with 177 (93.2%) reporting single status. Regarding housing, 160 students (84.2%) lived with their families, 24 (12.6%) lived alone, and only 6 (3.2%) lived with roommates.

**Table 1:** Demographic characteristics.

Items		n=190	%
Age group	<21	111	58.4
	≥21	79	41.6
Gender	Male	84	44.2
	Female	106	55.8
Marital status	Unmarried	177	93.2
	Married	13	6.8
Housing status	Living with family	160	84.2
	Living alone	24	12.6
	Living with roommates	6	3.2

Table 2 presents the distribution of participants across colleges and academic years. The College of Medicine had the largest group with 31 students (16.3%), followed by the College of Science with 30 (15.8%) and Applied Medical Sciences with 28 (14.7%). The largest academic year group comprised first-year students (46, 24.2%), followed by second-year (41, 21.6%) and fourth-year (35, 18.4%) students.

**Table 2:** College characteristics.

Items		n=190	%
College	Medicine	31	16.3
	Applied medical sciences	28	14.7
	Engineering	12	6.3
	Science	30	15.8
	Sharia and law	23	12.1
	Business administration	22	11.6
	Computers and information technology	14	7.4
	Applied college	8	4.2
	Education and arts	7	3.7
	Art and design	7	3.7
	Other	8	4.2
Year	First	46	24.2
	Second	41	21.6
	Third	22	11.6
	Fourth	35	18.4
	Fifth	9	4.7
	Sixth	30	15.8
	Internship	7	3.7

Table 3 examines the association between OCI-R scores and demographics. Among participants under 21 years of age, 47 (42.3%) showed probable OCD symptoms, compared to 32 (40.5%) among those aged 21 and older (p=0.882). Females showed a higher rate of probable OCD symptoms (48, 45.3%) than males (31, 36.9%) (p=0.300). Married participants had a slightly higher prevalence (6, 46.2%) compared to unmarried participants (73, 41.2%) (p=0.776). Those living alone (13, 54.2%) or with roommates (3, 50.0%) had higher rates than those living with family (63, 39.4%) (p=0.316), although none of these were statistically significant.

**Table 3:** Association between OCI-R score and demographic characteristics. OCD: obsessive-compulsive disorder; OCI-R: Obsessive-Compulsive Inventory-Revised. Chi-square and Fisher's exact tests were used. Statistical significance was set at p<0.05.

Items		Probability of OCD				p-value
		Non-probable OCD		Probable OCD		
		N=111	%	N=79	%	
Age group	<21	64	57.7	47	42.3	0.882
	≥21	47	59.5	32	40.5	
Gender	Male	53	63.1	31	36.9	0.300
	Female	58	54.7	48	45.3	
Marital status	Unmarried	104	58.8	73	41.2	0.776
	Married	7	53.8	6	46.2	
Housing status	Living with family	97	60.6	63	39.4	0.316
	Living alone	11	45.8	13	54.2	
	Living with roommates	3	50.0	3	50.0	

Table 4 explores the relationship between college-related variables and probable OCD. The highest rates were observed among engineering (7, 58.3%) and applied medical sciences students (16, 57.1%), while medical students had the lowest (8, 25.8%). Among academic year groups, fifth-year students had the highest prevalence (7, 77.8%), while sixth-year students had the lowest (7, 23.3%). These differences were not statistically significant (p=0.432 for college and p=0.104 for year).

**Table 4:** Association between OCI-R score and college characteristics. OCD: obsessive-compulsive disorder; OCI-R: Obsessive-Compulsive Inventory-Revised. Chi-square and Fisher's exact tests were used. Statistical significance was set at p<0.05.

Items		Probability of OCD				p-value
		Non-probable OCD		Probable OCD		
		N=111	%	N=79	%	
College	Medicine	23	74.2	8	25.8	0.432
	Applied medical sciences	12	42.9	16	57.1	
	Engineering	5	41.7	7	58.3	
	Science	18	60.0	12	40.0	
	Sharia and law	12	52.2	11	47.8	
	Business administration	14	63.6	8	36.4	
	Computers and information technology	8	57.1	6	42.9	
	Applied college	5	62.5	3	37.5	
	Education and arts	5	71.4	2	28.6	
	Art and design	3	42.9	4	57.1	
	Other	6	75.0	2	25.0	
Year	First	28	60.9	18	39.1	0.104
	Second	21	51.2	20	48.8	
	Third	14	63.6	8	36.4	
	Fourth	19	54.3	16	45.7	
	Fifth	2	22.2	7	77.8	
	Sixth	23	76.7	7	23.3	
	Internship	4	57.1	3	42.9	

## Discussion

In this study, probable OCD symptoms were slightly more prevalent in females (45.3%) than males (36.9%), although not statistically significant (p=0.300). Students living alone (54.2%) or with roommates (50.0%) had higher rates of probable OCD symptoms than those living with family (39.4%), but these differences were not statistically significant (p=0.316). Engineering and applied medical sciences students had the highest rates of probable OCD symptoms (58.3% and 57.1%), while medical students had the lowest (25.8%), with no significant association between college and OCD symptoms prevalence. Among academic year groups, fifth-year students had the highest prevalence of probable OCD symptoms (77.8%), while sixth-year students had the lowest (23.3%), although this association was not statistically significant.

According to a cross-sectional survey of four major Saudi Arabian medical universities, female OCI-R scores were significantly higher than those of males [15]. According to another meta-analysis of OCD prevalence, women were 1.6 times more likely than men to have OCD, with lifetime prevalence rates of 1.5% for women and 1.0% for men [16]. These results are in line with ours, which indicated a higher prevalence of OCD symptom severity in women. Research indicates that reproductive events in females can influence OCD symptom severity, with some women experiencing onset or exacerbation during pregnancy or postpartum periods [17]. ​According to a study done at an Iraqi medical institution, the prevalence of OCD symptoms was substantially correlated with younger age and earlier academic years [10]. Although our research supported the contention that probable OCD symptoms are more common in those under 21 years (42.3%), they also revealed that fifth-year students had a high prevalence of likely OCD symptoms. Another research investigation revealed that among individuals with OCD, 61.0% of the participants had early-onset OCD (less than 19 years old), and 39.0% had late-onset OCD (more than 20 years old) [18].

A study involving OCD patients reported that 51.7% of their participants were single and 41.1% were married. However, this study revealed that 42.2% of our participants with OCD symptoms were married. OCD significantly influences marital status and the quality of marital relationships. Research has underscored the importance of addressing OCD symptoms not only for the well-being of the individual but also for maintaining and improving marital relationships [19]. According to a study, before receiving therapy, about 50% of married OCD patients experience marital difficulties. Notably, it has been demonstrated that in these situations, behavioral therapy targeted at treating OCD symptoms reduces marital distress [20]. Another cross-sectional study evaluated that the severity of OCD symptoms negatively correlates with the marital adjustment of spouses. As OCD severity increases, spouses often report greater challenges in marital adjustment [21].

Although these differences were not statistically significant, this study demonstrates that students who lived alone (54.2%) had a higher incidence of probable OCD symptoms than those who lived with family (39.4%). They might experience higher levels of loneliness, potentially explaining the observed trend in the prevalence of OCD symptoms. According to a study conducted on 395 young adults, perceived loneliness was positively correlated with obsessive-compulsive symptoms (OCS), and this correlation persists even after adjusting for social anxiety and depression [22]. However, another study conducted among university students in Turkey revealed the significant association of OCD with students living in their parents' house and with verbal abuse in the family [23].

In our research, medical students had the lowest rate of likely OCD symptoms (25.8%), whereas engineering (58.3%) and applied medical sciences students (57.1%) had the highest rates. Using the OCI-R screening method, a study conducted at Umm Al-Qura University also revealed that 20% of applied medical science students who enrolled in the laboratory medicine department had significantly higher OCS [24]. Additionally, a comparative research study comparing medical and engineering students in North-East India found that medical students had much higher mean scores for psychopathologies than engineering students [25]. These results contradict the current study's findings, which show that medical students experience less OCD symptoms than engineering students.

This study has several limitations. One significant limitation is the small sample size, which may not fully represent the broader student population. This restricts the generalizability of the findings and may lead to biased estimates of the prevalence of OCD symptoms. Additionally, the cross-sectional design limits the ability to establish causal relationships between factors and OCD symptoms. Self-reporting in surveys can introduce self-bias, as participants may underreport or overreport their symptoms. It would be preferable to include a diagnostic evaluation by a psychiatrist or psychologist to confirm the diagnosis. Recall bias may also affect the accuracy of the data, as participants might not accurately remember past experiences related to OCD symptoms. Future studies with larger, more diverse samples and longitudinal designs are recommended to provide a more comprehensive understanding of the prevalence of OCD symptoms and its associated factors among university students.

The findings emphasize the need for targeted mental health programs in universities, particularly for students living away from family. Academic stressors may contribute to tendencies for OCD symptoms, requiring faculty-driven support initiatives. Institutions should implement counseling services, stress management workshops, and peer support programs to help students cope effectively. Understanding the diverse presentations of OCD symptoms across demographics can aid in early intervention. Future research should explore contributing factors such as academic workload, coping mechanisms, and social support. Longitudinal studies are needed to assess the long-term impact of stressors on OCD symptoms development, guiding interventions that promote student well-being, resilience, and academic success.

## Conclusions

This study explored the prevalence and distribution of OCD symptoms among university students in Tabuk, revealing notable differences across gender, living arrangements, academic programs, and year of study. The findings indicated that female students, those living alone, and students in specific programs such as Engineering and Applied Medical Sciences reported higher probable OCD symptom rates. Conversely, medical students and sixth-year students reported the lowest rates. These patterns suggest that both environmental and academic factors may influence the expression of OCD symptom in this population. The results revealed that females and students experiencing academic or social pressures may be more vulnerable to OCD symptoms. Importantly, these findings underline the need for proactive mental health strategies at the institutional level, particularly for identified high-risk groups. University counseling services, targeted awareness programs, and early screening could help identify students in need of support. Future research should build on these findings using larger, more diverse samples and longitudinal designs. Incorporating clinical diagnostic assessments by mental health professionals is also recommended to validate self-reported symptoms and improve diagnostic accuracy.

## Appendices

**Table 5:** OCI-R. The OCI-R is a short version of the OCD. OCD: obsessive-compulsive disorder; OCI-R: Obsessive-Compulsive Inventory-Revised.

Do you agree to participate in this questionnaire?
- Yes
- No
This section is about demographic data:
Are you a student in Tabuk University?
- Yes
- No
Age
- 18
- 19
- 20
- 21
- 22
- 23
- 24
- 25
- 26
Gender
- Male
- Female
Marital status
- Single
- Married
Housing status
- Living with family
- Living alone
- Living with roommate
University major
- Medicine
- Dentistry
- Pharmacology
- Applied medical science
- Nursing
- Engineering
- Computer and information technology
- Science
- Business administration
- Education and arts
- Sharia and law
- Art and design
- Others
Academic year
- First year
- Second year
- Third year
- Fourth year
- Fifth year
- Sixth year
- Internship
The following statements refer to experiences that many people have in their everyday lives. Circle the number that best describes how much that experience has distressed or bothered you during the past month:
I have saved up so many things that they get in the way
- Not at all
- A little
- Moderately
- A lot
- Extremely
I check things more often than necessary
- Not at all
- A little
- Moderately
- A lot
- Extremely
I get upset if objects are not arranged properly
- Not at all
- A little
- Moderately
- A lot
- Extremely
I feel compelled to count while I am doing things
- Not at all
- A little
- Moderately
- A lot
- Extremely
I find it difficult to touch an object when I know it has been touched by strangers or certain people
- Not at all
- A little
- Moderately
- A lot
- Extremely
I find it difficult to control my own thoughts
- Not at all
- A little
- Moderately
- A lot
- Extremely
I collect things I don’t need
- Not at all
- A little
- Moderately
- A lot
- Extremely
I repeatedly check doors, windows, drawers, etc.
- Not at all
- A little
- Moderately
- A lot
- Extremely
I get upset if others change the way I have arranged things
- Not at all
- A little
- Moderately
- A lot
- Extremely
I feel I have to repeat certain numbers
- Not at all
- A little
- Moderately
- A lot
- Extremely
I sometimes have to wash or clean myself simply because I feel contaminated
- Not at all
- A little
- Moderately
- A lot
- Extremely
I am upset by unpleasant thoughts that come into my mind against my will
- Not at all
- A little
- Moderately
- A lot
- Extremely
I avoid throwing things away because I am afraid I might need them later
- Not at all
- A little
- Moderately
- A lot
- Extremely
I repeatedly check gas and water taps and light switches after turning them off
- Not at all
- A little
- Moderately
- A lot
- Extremely
I need things to be arranged in a particular way
- Not at all
- A little
- Moderately
- A lot
- Extremely
I feel that there are good and bad numbers
- Not at all
- A little
- Moderately
- A lot
- Extremely
I wash my hands more often and longer than necessary
- Not at all
- A little
- Moderately
- A lot
- Extremely
I frequently get nasty thoughts and have difficulty in getting rid of them
- Not at all
- A little
- Moderately
- A lot
- Extremely
